# Sofosbuvir induced leucocytoclasic vasculitis: a case report

**DOI:** 10.1186/s12895-019-0086-4

**Published:** 2019-05-17

**Authors:** Elena Campione, Annunziata Dattola, Monia Di Prete, Daniele Di Paolo, Amedeo Ferlosio, Augusto Orlandi, Luca Bianchi

**Affiliations:** 1 Department of Dermatology, University of Rome Tor Vergata, Viale Oxford, 81, 00133 Rome, Italy; 2 Department of Anatomic Pathology, University of Rome Tor Vergata, Viale Oxford, 81, 00133 Rome, Italy; 3 Hepatology Unit, Department of Internal Medicine, Rome, Italy

**Keywords:** Sofosbuvir, Hepatitis C, Leukocytoclasic vasculitis

## Abstract

**Background:**

We describe a case of leucocytoclasic vasculitis induced by Sofosbuvir and its disappearence after the end of the therapy. The hepatitis C virus, firstly described in 1989, is a major global health problem, with high morbidity and mortality. We observed a temporal relationship between the treatment and the onset of vasculitis. We emphasize the multidisciplinary approach to the patients with liver disease to improve the quality of life of these patients.

**Case presentation:**

A 53-year-old Caucasian man with a history of hepatitis C virus genotype 1 infection was examined at our Department of Dermatology for the occurrence of palpable purpura. The patient referred that the first appearance of the dermatoses was about one month after initiation of therapy with Sofosbuvir for hepatitis C.

**Conclusions:**

Vasculitis appeared after the beginning of Sofosbuvir and, even though it was treated with different medications proved to be effective, it disappeared only after the conclusion of the therapy, giving a strong evidence to be a drug eruption.

## Background

Firstly described in 1989, hepatitis C virus (HCV), is now a leading cause of liver cirrhosis and, subsenquently, of hepatocellular carcinoma. [1]

Blood is the main means of transmission of HCV as in transfusion, injection drug use, organ transplantation, hemodialysis, or accidental exposure; however, other ways have also been documented as unprotected sexual contact and vertical mother-to-child transmissions. [2, 3]

The World Health Organization (WHO) reported that 3–4 million people are newly infected by HCV per year and 130–170 million people are chronically infected. Over 350,000 people/year die for hepatitis C-related liver diseases. [4] These data lead to the conclusion that hepatitis C is a global health problem.

HCV is classified into seven genotypes with multiple subtypes on the basis of phylogenetic and sequence analyses of whole viral genomes. [1–7] Different genotypes diverge at 30–35% of nucleotide sites. Strains that belong to the same subtype differ at < 15% of nucleotide sites. [6]

Until 2011, the standard therapy to treat HCV infection was the combination of pegylated interferon (PegIFN)-alpha and ribavirin (RBV) for 24 or 48 weeks.

After 2011, new oral effective drugs have been introduced to treat chronic HCV infections, with cure rate of about 90%. They opened a new era in the management of chronic infections after 25 years from the discovery of the virus. They are called directly-acting antiviral agents (DAAs).

Now, there is a new generation of DAAs. Sofosbuvir (SOF), simeprevir (SIM) and daclatasvir (DCV) are included in this group. These drugs increase the sustained virologic response (SVR) rate with fewer side effects and short duration of treatment. [8] SOF is one of the most promising antiviral therapies with high SVR rate of the HCV (> 90%). DAAs are administered with or without PegIFN and/or RBV, with different duration of treatment according to the combination used. The optimal regimen in IFN eligible patients is 12 weeks of PegIFN and RBV plus SOF/SIM/DCV, but in IFN ineligible patients, it is recommended a 24 weeks course of SOF-RBV or 12 weeks of SOF/SIM or SOF/DCV with or without RBV. [9]

SOF is nucleotide analogue inhibitor of the HCV NS5B RNA-polymerase. It is active against all the genotypes. It has to be taken orally at the dose of 400 mg once daily, without relation to food intake. [10]

## Case presentation

**Fig. 1:**
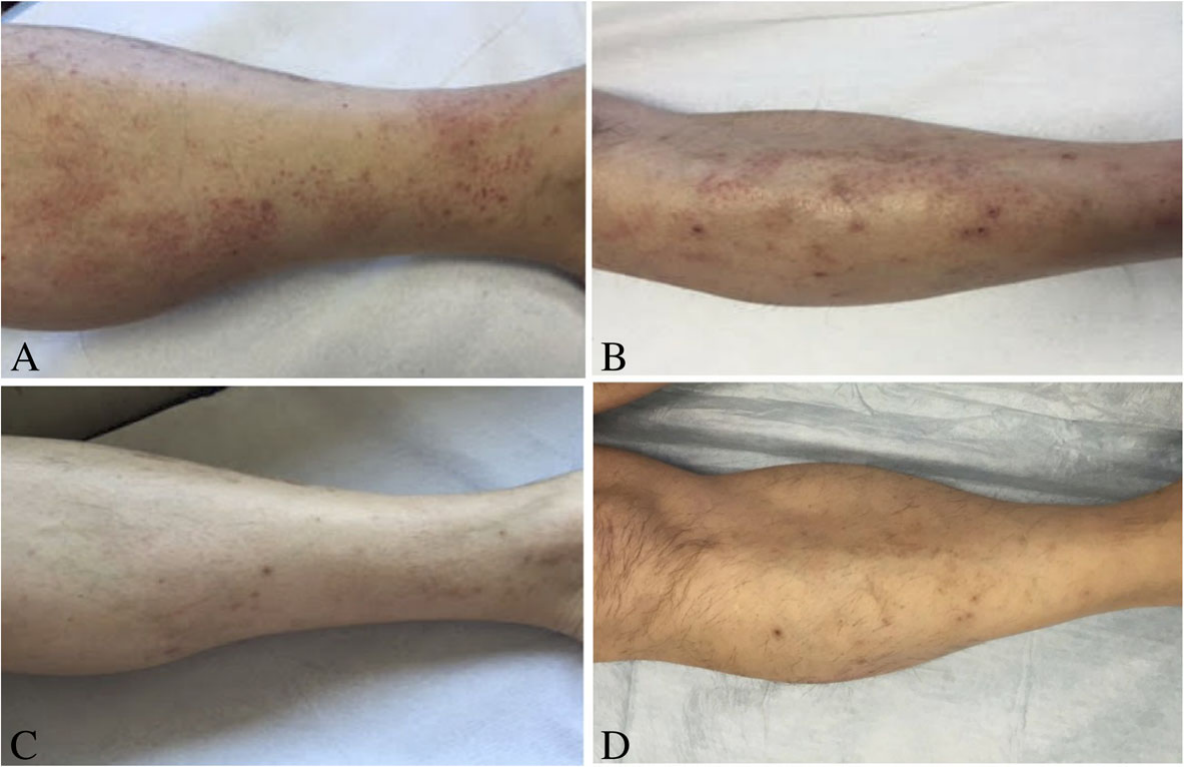
**a**- **b**) Diffuse erythematous maculaes and papules on the lower extremities at the baseline. **c**-**d**) Disappearence of the lesions one month after discontinuation of the therapy with Sofosbuvir

**Fig. 2:**
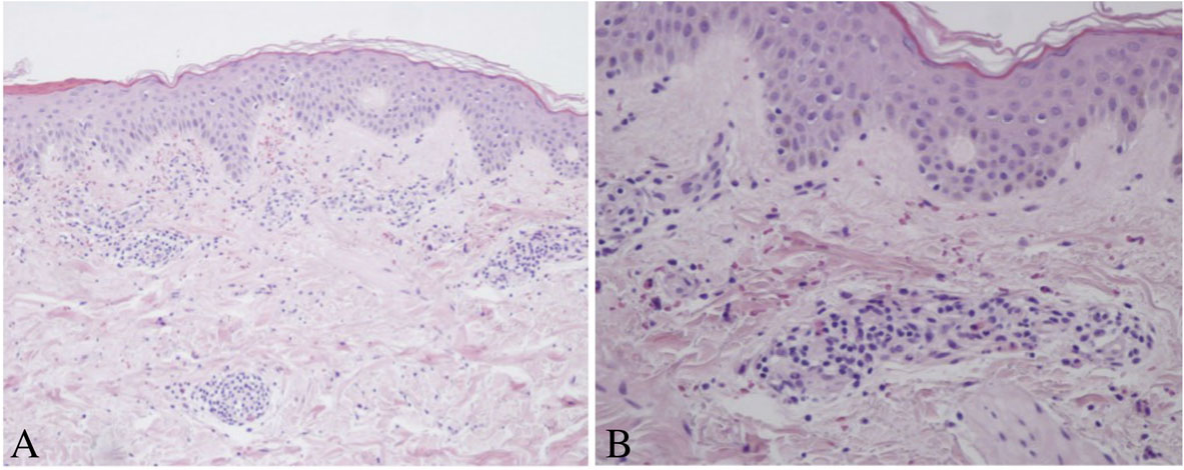
**a**) Representative histological picture showing perivascular inflammatory infiltrate in papillary and mid dermis associated to erythrocyte extravasation and mild dermal oedema. The epidermis showed only focal spongiosis and basal vacuolization (Hematoxylin-Eosin, Original magnification 100x). **b**) At higher magnification, the inflammatory cells were predominantly composed by small lymphocytes, hystiocites nd eosinophils around and within capillary vessels (Hematoxylin-Eosin, Original magnification 200x)

We report a case of 53-year-old Caucasian man with a history of hepatitis, HCV genotype 1-related. He referred to our Dermatology Department for the occurrence of palpable purpura. Erythematous maculae and papules were widespread on trunk and lower extremities associated with pain, burning and itching (Fig. 1, A and B). The patient referred that the first appearance of the dermatoses was about one month from the beginning of the therapy for his hepatitis. The patient was ineligible for the treatment with IFN, so he began a 24-weeks course of SOF 400 mg/daily for 24 weeks. Skin lesions were evaluated by dermoscopy (Dermlite Foto, 3Gen, Dana Point, California, USA) and the examination revealed a polymorphous vascular pattern, surrounded by a subtle erythematous border. A 4-mm punch biopsy of a lesion from the leg was performed. Microscopically, at low magnification, skin showed perivascular inflammatory infiltrate in papillary and mild dermis associated to erythrocyte extravasation and mild dermal oedema (Fig. 2A). The epidermis showed only focal spongiosis and basal vacuolization. At higher magnification, the inflammatory cells were predominantly composed by small lymphocytes, histiocytes and eosinophils around and within capillary vessel walls with endothelial swelling (Fig. 2B). The eosinophils were more that 5 per 10 high-power fields. According to the histological aspect, a diagnosis of drug-induces lymphocytic small vessel vasculitis was expressed. In fact, one of the main cause of increased tissue eosinophil count is the hypersensitivity reaction to a drug or immunotherapic agent, as described by Bahrami et al. [11]

Topical corticosteroids and emollient were prescribed to the patient, but, after an initial improvement, he came again to our observation for relapse and worsening of the dermatoses.

Blood analysis revealed no substantial alterations, excepting for 1.20% of crioglobuline (normal range 0.00–0.40) and anti-nucleus antibodies (ANA) positivity 1:80. No positivity was found for anti-neutrophil cytoplasmic antibodies (ANCA).

It was our opinion that the therapy triggered a vasculitis-like drug eruption. Based on our clinical observations, we assumed a causal link between skin lesions and medication, as it was demonstrated by the resolution of vasculitic lesions one month after the discontinuation of the treatment (Fig. 1, C and D).

## Discussion and conclusion

Leucocytoclasic vasculitis is a cutaneous small-vessel necrotizing vasculitis, with predominantly neutrophilic infiltrate, frequently associated with viral infections, comprised HCV. Many drugs are implicated in the pathogenesis as it is demonstrated for amoxicillin, phenobarbital, penicillin, rifampicin, diazepam and cephalosporin. [7, 8] Evaluating patients serum in these cases, it is common to confirm cryoglobulinemia. Cryoglobulins consist in polyclonal IgG with monoclonal (type II) or polyclonal (type III) IgM with rheumatoid factor (RF) activity. [9] In hepatitis C, HCV interacts with lymphocytes, modulating their function. It results in polyclonal B-cells activation, esiting in production of IgM with RF activity. Cutaneous manifestations generally resolve with viral count decreasing, during antiviral therapy. On the contrary, patients with constant high viremia and who relapses HCV infection, usually develop vasculitis again. [8, 9, 12, 13]

In literature, side effects in the use of SOF were reported only when it is administered in combination with Peg-IFN and/or RBV. [14] FISSION and NEUTRINO trials (phase 3 clinical trials that evaluate respectively the combination SOF-RBV and SOF-RBV plus Peg-IFN) reported a non-specified rash in 18% of treated patients, in addition to flu-like symptoms, fatigue and anemia, which are common during treatment with Peg-IFN and RBV. POSITRON and FUSION trials (two other phase 3 clinical trials, which consider the combination SOF-RBV) reported cutaneous rash at a rate similar to what seen in the placebo group. [14–16] More recently, it were described ANCA-associated vasculities (AAV) leading to a crescentic allograft glomerulonephritis after SOF-RBV therapy in a patient with kidney transplant and several cases (85/3000 patients) of AAV skin lesions without renal involvement. [17, 18] On the contrary, in our case the patient did not develop ANCA, but a moderate crioglobulinaemia and positivity to serum ANA.

Although it has been repeatedly demonstrated the efficacy and safety of SOF in HCV infection therapy, [15, 16] we observed a temporal relationship between the treatment and the onset of vasculitis. It appeared after one month from the beginning of SOF and, even though it was treated with medications proved to be effective in cutaneous vasculitis, it disappeared only after discontinuation of the therapy, giving a strong evidence to be a drug eruption.

Therefore, we emphasize the multidisciplinary approach to the patients with liver disease that can have acute and chronic dermatoses related to the drug that can be controlled by improving the quality of life of these patients.

## Abbreviations

AAV: ANCA-associated vasculities
ANA: Anti-nucleus antibodies
ANCA: Anti-neutrophil citoplasmic antibodies
DAAs: Directly-acting antiviral agents
DCV: Daclatasvir
HCV: Hepatitis C virus
PegIFN: Pegylated interferon
RBV: Ribavirin
RF: Rheumatoid factor
SIM: Simeprevir
SOF: Sofosbuvir
SVR: Sustained virologic response
WHO: World Health Organization
